# Supplementary material for: Solution structure of human myeloid-derived growth factor suggests a conserved function in the endoplasmic reticulum
Source: Nat Commun. 2019 Dec 9;10:5612. doi: 10.1038/s41467-019-13577-5 (PMC6901522; doi:10.1038/s41467-019-13577-5)
Supplement: Supplementary file 1 — Supplementary Information [file 41467_2019_13577_MOESM1_ESM.pdf]

Supplementary Information

Solution structure of human myeloid-derived growth factor suggests a conserved function in the endoplasmic reticulum

Valeriu Bortnov et al.

Supplementary Table 1. Compilation of recombinant hMYDGF CD spectra characteristics

Recombinant Protein	CD spectra maxima and minima[a] (nm)			Secondary structure composition[b] (%)		
	Maximum	Minimum 1	Minimum 2	β-sheet	α-helix	Irregular
Insect cell-derived hMYDGF (C-terminal 6xHis-tag; n = 3)	201 ± 1	218 ± 1	232 ± 1	38 ± 1	2 ± 0	60 ± 1
Bacteria-derived hMYDGF (N-terminal 6xHis-tag; n = 4)	202 ± 0	218 ± 1	230 ± 3	42 ± 1	2 ± 0	56 ± 1
Bacteria-derived hMYDGF (cleaved 6xHis-tag; n = 8)	202 ± 1	219 ± 1	234 ± 2	45 ± 2	0 ± 0	54 ± 2
NMR structure of hMYDGF (bacteria-derived; cleaved 6xHis-tag)	-	-	-	48	2	50

[a]All CD spectra were collected at 25°C in buffers ranging from pH 3.7-7.5 and subtracted from their corresponding buffer spectra. The values reported are averages from multiple spectra (n), with standard deviations reported after each value. [b]The BeStSel server[1] was used to predict the secondary structure content for the recombinant proteins based on the CD spectra. Source data for all spectra are provided as a Source Data file.

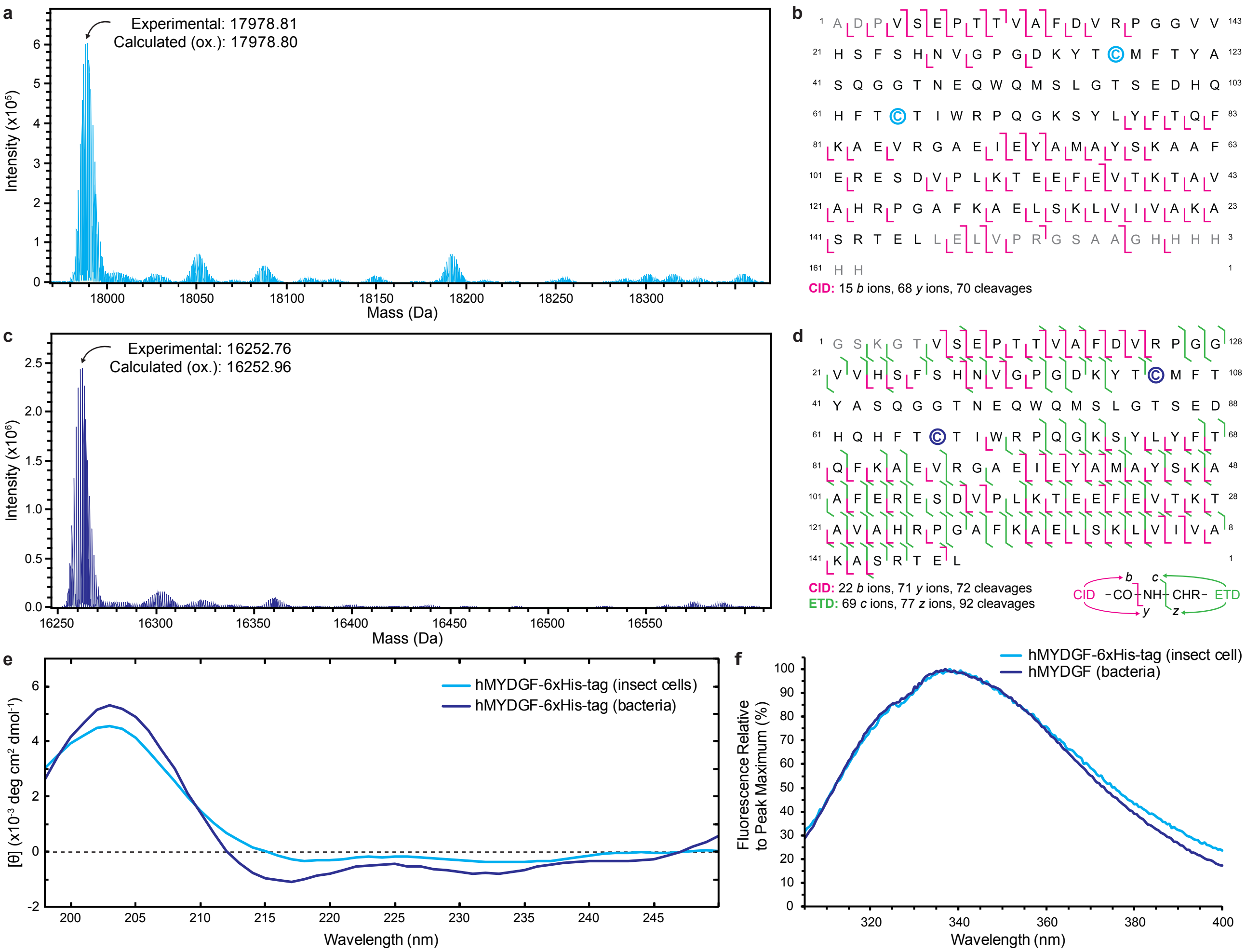

Supplementary Figure 1. Comparison of insect cell- and bacteria-derived hMYDGF. a. High resolution, deconvoluted MS spectrum of insect cell-derived hMYDGF (C-terminal 6xHis-tag) and **b.** its corresponding MS/MS CID (magenta) fragmentation map. **c.** Deconvoluted MS spectrum of bacteria-derived hMYDGF (cleaved 6xHis-tag) and **d.** its corresponding MS/MS CID (magenta) and ETD (green) fragmentation map. Experimental monoisotopic masses of both protein constructs matched their calculated masses with cysteines in their oxidized forms (S-S). No ion fragments were found between the two cysteines (circled) of either protein, providing evidence that hMYDGF forms a disulfide bond in both expression systems. Residues non-native to mature hMYDGF are colored gray in the fragmentation maps. **e.** Smoothed CD spectra of hMYDGF secreted from baculovirus-infected High Five insect cells with a C-terminal 6xHis-tag (light blue) and hMYDGF purified and refolded from *E. coli* lysate with a N-terminal 6xHis-tag (dark blue) overlay well with one another. Scans are representative of those collected at least 3 times at 25°C with buffer baseline scans subtracted. **f.** Intrinsic tryptophan fluorescence spectra of insect cell- (C-terminal 6xHis-tag; light blue) and bacteria-derived (lacking 6xHis-tag; dark blue) hMYDGF displayed as a percentage relative to the fluorescence maxima of 338.5 nm and 338.0, respectively. The curves are averages of 3 scans with subtraction of buffer baselines. Source data for CD and tryptophan fluorescence experiments are provided as a Source Data file.

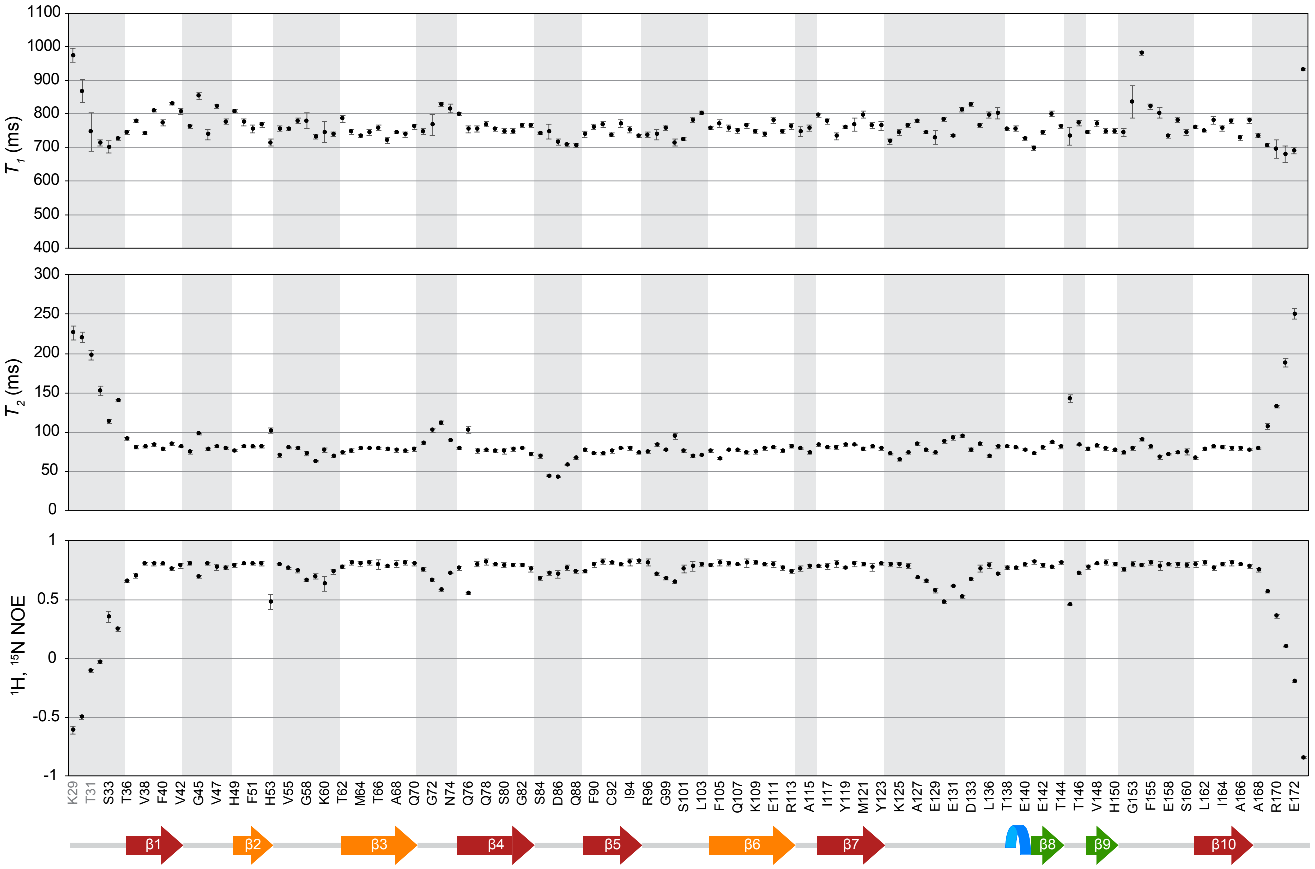

Supplementary Figure 2. ^{15}N T_1 and T_2 relaxation times and ^{1}H, ^{15}N NOE of hMYDGF residues. Residues involved in secondary structure had average T_1 and T_2 values of 763.6 ms and 78.6 ms, respectively. These average relaxation times were used to estimate an overall correlation time (τ_c) of 9.4 ns for hMYDGF. Fluctuation in ^{15}N T_1, T_2, and heteronuclear NOE from these average values align well with regions of the hMYDGF backbone that lack regular secondary structure (gray blocks). Residues introduced by cloning are in gray and numbered relative to V32, the first residue of mature hMYDGF. The heteronuclear NOE experiments were conducted in duplicate, with the data presented here as the average and the error bars displaying the maximum and minimum experimental values. The ^{15}N T_1 and T_2 values were calculated by NMRFAM-SPARKY[2] through an exponential fit of the experimental data with error bars reflecting the likely error of the best fit from the true T values caused by Gaussian noise in the peak height values.

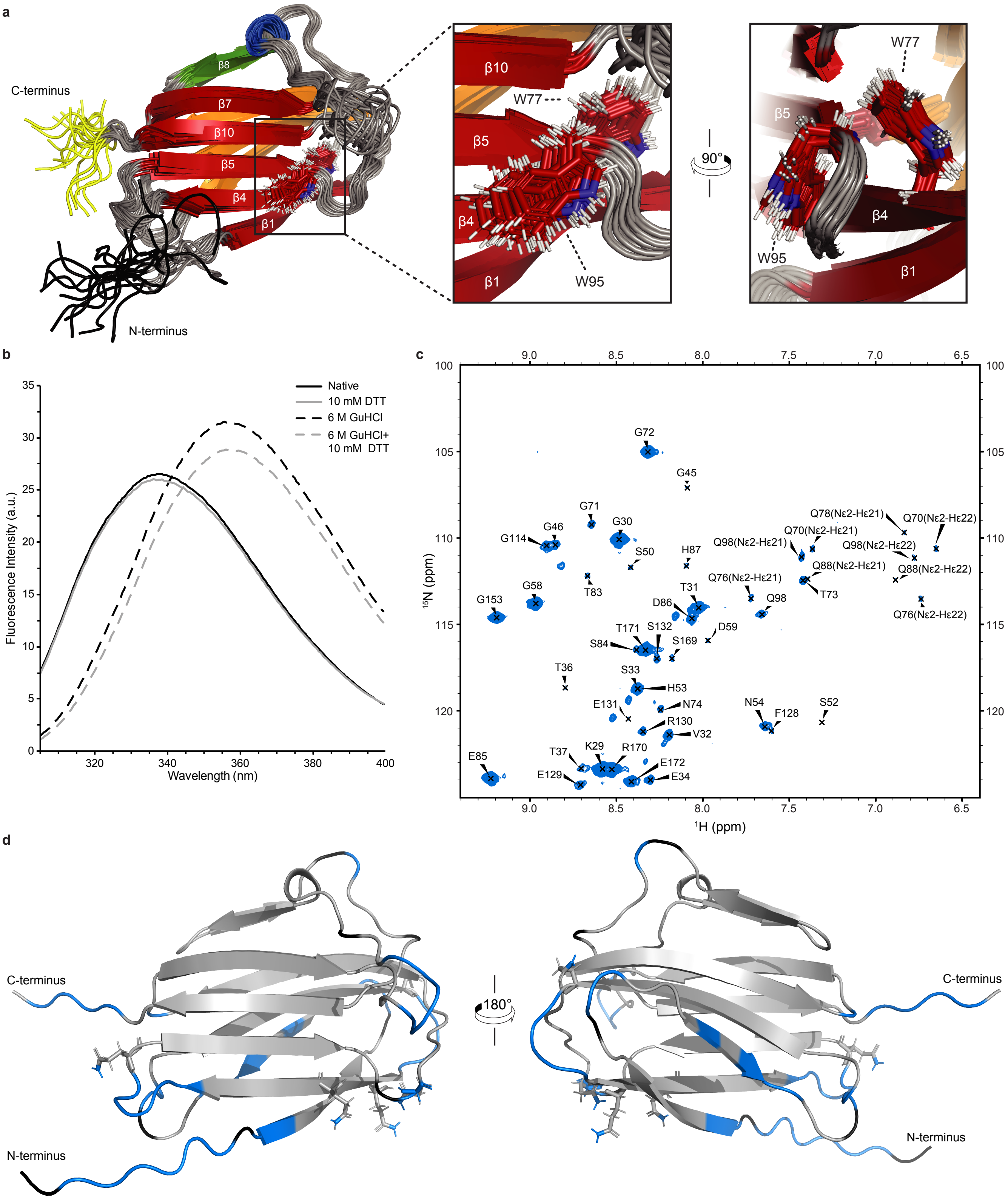

a
C-terminus
N-terminus
β8
β7
β10
β5
β4
β1
W77
W95
90°
b
Native
10 mM DTT
6 M GuHCl
6 M GuHCl+
10 mM DTT
Fluorescence Intensity (a.u.)
Wavelength (nm)
0
5
10
15
20
25
30
35
320
340
360
380
400
c
9.0
8.5
8.0
7.5
7.0
6.5
100
105
110
115
120
^{15}N (ppm)
^{1}H (ppm)
G72
G45
G71
G30
G46
G114
S50
H87
T83
G58
G153
T31
D86
Q98
D59
T171
S132
S84
S169
T36
S33
H53
E131
N74
R130
V32
N54
F128
S52
T37
K29
R170
E85
E129
E172
E34
T73
Q78(Nε2-Hε21)
Q70(Nε2-Hε22)
Q70(Nε2-Hε21)
Q98(Nε2-Hε21)
Q98(Nε2-Hε22)
Q88(Nε2-Hε21)
Q88(Nε2-Hε22)
Q76(Nε2-Hε21)
Q76(Nε2-Hε22)
d
C-terminus
N-terminus
180°

Supplementary Figure 3. Characterization of the two hMYDGF tryptophan residues. a. hMYDGF residues W77 (core-oriented) and W95 (solvent-oriented) are located in close proximity, with little spatial variability among the 20 most energetically-stable conformers. **b.** Intrinsic tryptophan fluorescence of native hMYDGF in the absence (black, solid) or presence (gray, solid) of 10 mM DTT had overlaying spectra after excitation at 290 nm. Denaturation of hMYDGF in 6 M GuHCl without (black, dotted) or with 10 mM DTT (gray, dotted) resulted in a red-shift of the emission peak from 338.0 to 357.0 nm with increased fluoresce intensity. Spectra are an average of 3 scans and have been baseline subtracted. Source data for each spectra are provided as a Source Data file. **c.** hMYDGF hydrogen exchange with water was monitored by recording clean SEA HSQC spectra with mixing times ranging from 10-140 ms. The clean SEA HSQC spectrum acquired with a 140 ms mixing time is presented here with assigned peaks of hydrogen-exchanged backbone amides and side-chains. Peaks corresponding to the two hMYDGF tryptophans were not observed. **d.** Residues corresponding to the assigned peaks in panel **c** are highlighted in blue in the hMYDGF structure. Residues with side-chain amides in the clean SEA HSQC spectrum are represented as sticks. Unobserved backbone amide resonances for which assignments were known are in gray. Prolines, the two N-terminal residues with unassigned backbone amide resonances, and A157 that was not observed in these spectra are in black.

Supplementary Table 2. Structural statistics for hMYDGF/cKDELR2 complexes modeled by HADDOCK

Cluster statistics	Cluster 1[a]	Cluster 2[a]
HADDOCK score (arbitrary units)	-73.2 ± 0.6	-94.9 ± 3.6
Cluster size (# models)	203	197
RMSD from the overall lowest-energy structure (Å)	11.6 ± 0.2	0.8 ± 0.5
hMYDGF residues within 5 Å of cKDELR2 (lowest-energy model)	16	14
cKDELR2 residues within 5 Å of hMYDGF (lowest-energy model)	31	35
Polar contacts between hMYDGF and cKDELR2 (lowest-energy model)	11	10
Salt bridges between hMYDGF and cKDELR2 (lowest-energy model)	2	2

[a]The computational docking program HADDOCK[3–5] was used to generate 400 complexes grouped into two clusters between the lowest-energy NMR conformer of hMYDGF and the crystal structure of cKDELR2 (6I6H[6]; 96% sequence identity with human KDELR2) both solved at pH 6. The lowest-energy complexes from each of the two clusters are presented in Supplementary Figure 4.

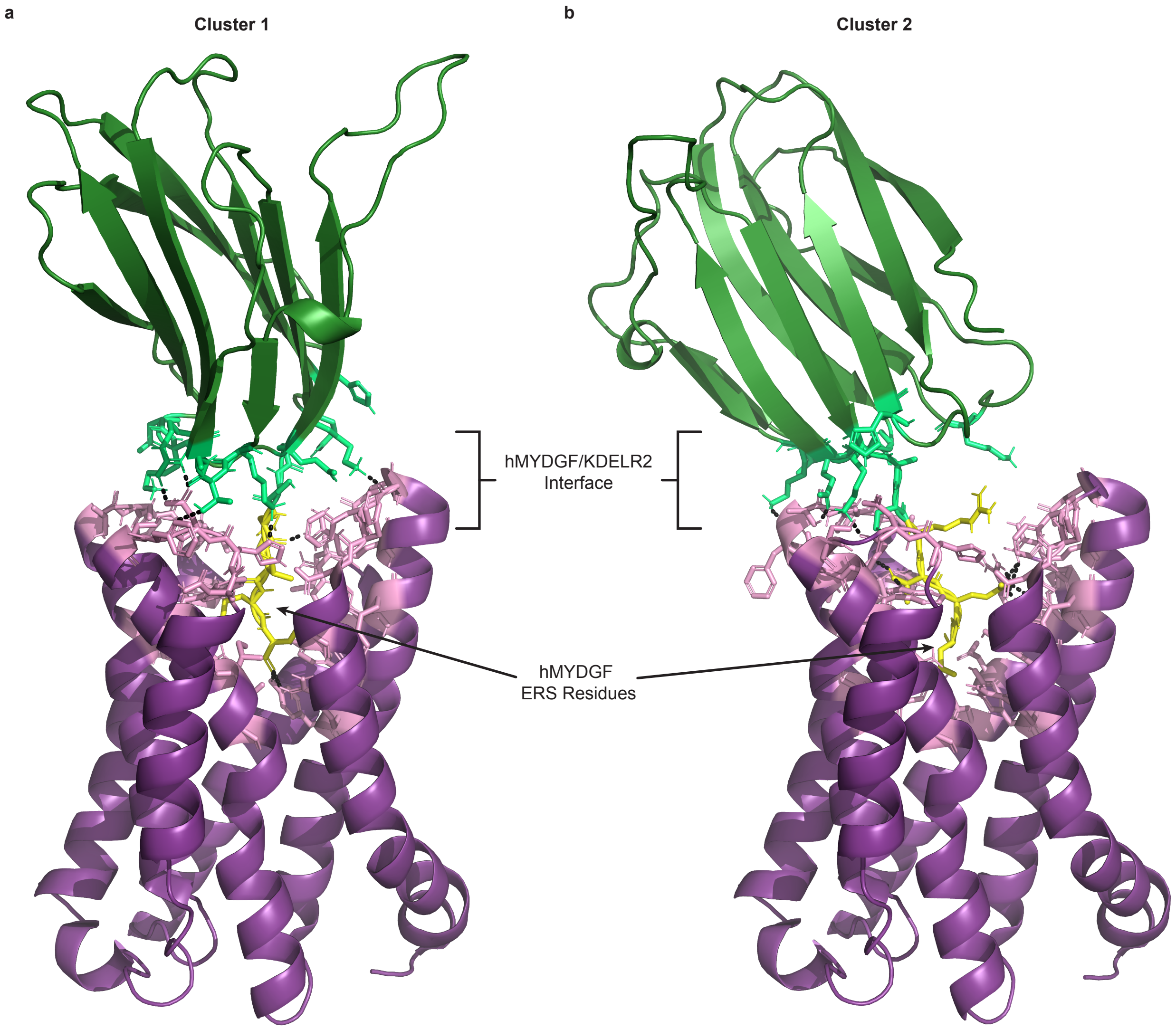

Supplementary Figure 4. hMYDGF docked onto cKDELR2. a. The lowest-energy complex from cluster 1 and **b.** cluster 2 of hMYDGF (green) docked onto cKDELR2 (6I6H[6]; purple) using HADDOCK[3–5] (see Supplementary Table 2 for cluster statistics). hMYDGF is less aligned with the cKDELR2 cavity and is rotated ~130° around the ERS (residues RTEL, yellow) in cluster 2 relative to cluster 1. In both models, the hMYDGF ERS binds a pocket in cKDELR2. The remaining hMYDGF residues within 5 Å of cKDELR2 (light green) and the cKDELR2 residues within 5 Å of hMYDGF (pink) are depicted as sticks to reveal an interface between the two proteins. Numerous putative polar contacts (dashed, black lines) were present between these close-proximity residues in the binding pockets and at the interfaces of the two proteins.

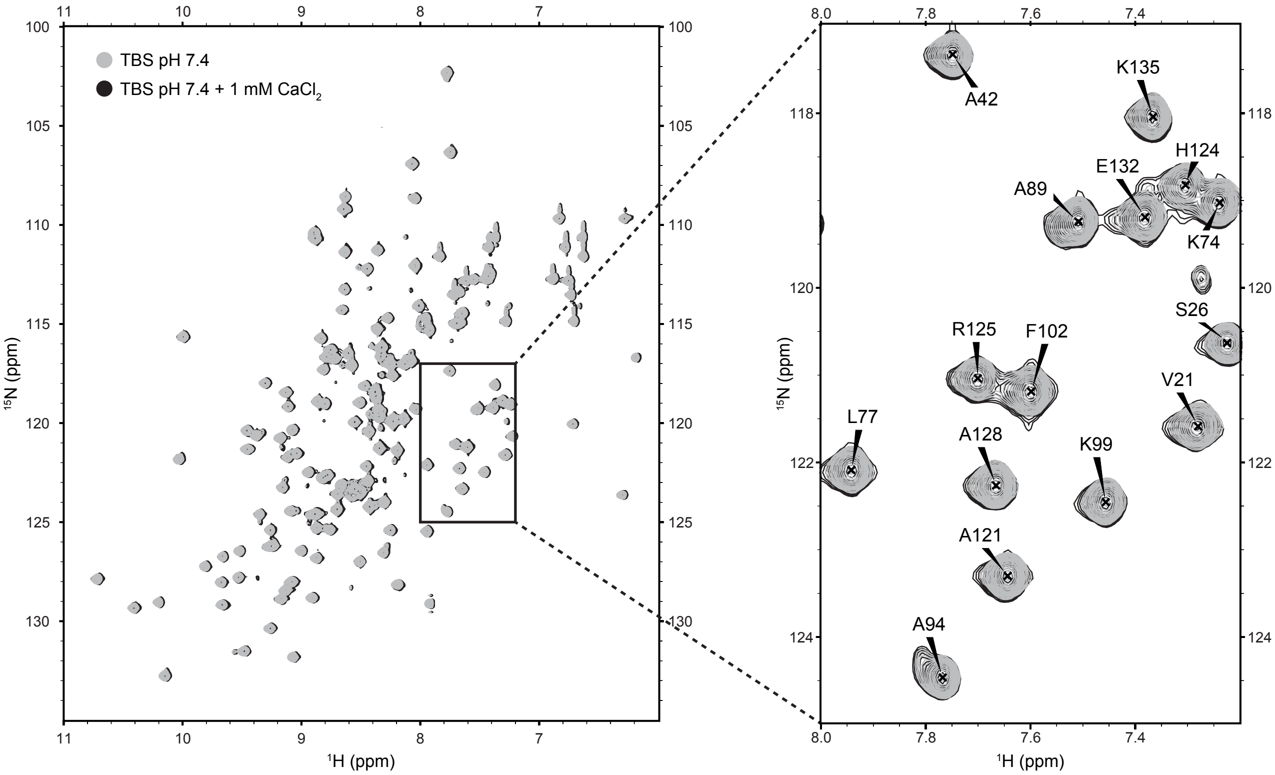

Supplementary Figure 5. Effect of added calcium ion on the NMR spectrum of hMYDGF. ^{1}H, ^{15}N HSQC spectra of hMYDGF were collected in the absence and presence of 0.5-, 1-, 2-, and 4-fold molar excess of calcium. The ^{1}H, ^{15}N HSQC spectrum of the highest calcium condition (1 mM $CaCl_2$) is presented here (black), which overlays completely with the spectrum of hMYDGF lacking calcium (gray).

Supplementary References

1. Micsonai, A. *et al.* BeStSel: a web server for accurate protein secondary structure prediction and fold recognition from the circular dichroism spectra. *Nucleic Acids Res.* **46**, W315–W322 (2018).
2. Lee, W., Tonelli, M. & Markley, J. L. NMRFAM-SPARKY: enhanced software for biomolecular NMR spectroscopy. *Bioinformatics* **31**, 1325–1327 (2015).
3. Dominguez, C., Boelens, R. & Bonvin, A. M. J. J. HADDOCK: A Protein−Protein Docking Approach Based on Biochemical or Biophysical Information. *J. Am. Chem. Soc.* **125**, 1731–1737 (2003).
4. de Vries, S. J. *et al.* HADDOCK versus HADDOCK: New features and performance of HADDOCK2.0 on the CAPRI targets. *Proteins Struct. Funct. Bioinforma.* **69**, 726–733 (2007).
5. van Zundert, G. C. P. *et al.* The HADDOCK2.2 Web Server: User-Friendly Integrative Modeling of Biomolecular Complexes. *J. Mol. Biol.* **428**, 720–725 (2016).
6. Bräuer, P. *et al.* Structural basis for pH-dependent retrieval of ER proteins from the Golgi by the KDEL receptor. *Science.* **363**, 1103–1107 (2019).
