# Supplementary material for: Solution structure of human myeloid-derived growth factor suggests a conserved function in the endoplasmic reticulum
Source: Nat Commun. 2019 Dec 9;10:5612. doi: 10.1038/s41467-019-13577-5 (PMC6901522; doi:10.1038/s41467-019-13577-5)
Supplement: Supplementary file 3 — Reporting Summary [file 41467_2019_13577_MOESM3_ESM.pdf]

nature research

Corresponding author(s): Deane F. Mosher

Last updated by author(s): Nov 6, 2019

Reporting Summary

Nature Research wishes to improve the reproducibility of the work that we publish. This form provides structure for consistency and transparency in reporting. For further information on Nature Research policies, see Authors & Referees and the Editorial Policy Checklist .

Statistics

For all statistical analyses, confirm that the following items are present in the figure legend, table legend, main text, or Methods section.

n/a	Confirmed	
☐	☒	The exact sample size (*n*) for each experimental group/condition, given as a discrete number and unit of measurement
☐	☒	A statement on whether measurements were taken from distinct samples or whether the same sample was measured repeatedly
☒	☐	The statistical test(s) used AND whether they are one- or two-sided<br>*Only common tests should be described solely by name; describe more complex techniques in the Methods section.*
☒	☐	A description of all covariates tested
☐	☒	A description of any assumptions or corrections, such as tests of normality and adjustment for multiple comparisons
☐	☒	A full description of the statistical parameters including central tendency (e.g. means) or other basic estimates (e.g. regression coefficient) AND variation (e.g. standard deviation) or associated estimates of uncertainty (e.g. confidence intervals)
☒	☐	For null hypothesis testing, the test statistic (e.g. *F*, *t*, *r*) with confidence intervals, effect sizes, degrees of freedom and *P* value noted<br>*Give P values as exact values whenever suitable.*
☒	☐	For Bayesian analysis, information on the choice of priors and Markov chain Monte Carlo settings
☒	☐	For hierarchical and complex designs, identification of the appropriate level for tests and full reporting of outcomes
☒	☐	Estimates of effect sizes (e.g. Cohen's *d*, Pearson's *r*), indicating how they were calculated

Our web collection on statistics for biologists contains articles on many of the points above.

Software and code

Policy information about availability of computer code

Data collection	Nuclear magnetic resonance: TopSpin 3.5, VNMRJ. Amino acid conservation: ConSurf, Jalview. Extinction coefficient prediction: ExPASy ProtParam. Concentration determination: SoftMax Pro v6.3. Structure similarity: Dali Server. Protein cavity detection: CASTp. Protein docking: HADDOCK 2.2. Surface charge distribution: PDB2PQR, APBS, PROPKA3.1.
Data analysis	Mass spectrometry: Bruker DataAnalysis 4.3, MASH Suite Pro. Circular Dichroism spectroscopy: Igor Pro v6, CAPITO, BeStSel. Intrinsic tryptophan fluorescence: Datamax v2. Graphing and trendlines: Microsoft Excel. Nuclear magnetic resonance and structure: NMRPipe, NMRFAM-SPARKY, I-PINE, ARECA, PONDEROSA-C/S package, wwPDB Validation Service, MolProbity, PyMOL. Amino acid conservation: ConSurf, Jalview, iTOL.

For manuscripts utilizing custom algorithms or software that are central to the research but not yet described in published literature, software must be made available to editors/reviewers. We strongly encourage code deposition in a community repository (e.g. GitHub). See the Nature Research guidelines for submitting code & software for further information.

Data

Policy information about availability of data

All manuscripts must include a data availability statement. This statement should provide the following information, where applicable:

- Accession codes, unique identifiers, or web links for publicly available datasets
- A list of figures that have associated raw data
- A description of any restrictions on data availability

The solution structure of hMYDGF along with structural restraints was deposited in the PDB under accession code 6O6W. NMR data were deposited in BMRB under accession number 30584. MYDGF and vanin base protein families can be viewed in Pfam under accession codes PF10572 and PF19018, respectively. The lowest-energy hMYDGF/cKDELR2 complexes from HADDOCK cluster 1 and 2 were deposited in PDB-Dev under accession code PDBDEV_00000036. The source data underlying Figures 2a, 3a, c, Supplementary Table 1, and Supplementary Figures 1e-f, and 3b are provided as a Source Data file. The data that support the findings of this study are available from the corresponding author upon reasonable request.

Field-specific reporting

Please select the one below that is the best fit for your research. If you are not sure, read the appropriate sections before making your selection.

☒ Life sciences ☐ Behavioural & social sciences ☐ Ecological, evolutionary & environmental sciences

For a reference copy of the document with all sections, see nature.com/documents/nr-reporting-summary-flat.pdf

Life sciences study design

All studies must disclose on these points even when the disclosure is negative.

Sample size	Not applicable, the study focuses on the structural characterization of one protein.
Data exclusions	No data were excluded.
Replication	Circular dichroism and tryptopan fluorescence experiments were replicated successfully at least 3 times. Mass spectrometry was conducted on the insect cell derived construct once and twice with two different methods on the bacterially-derived construct. hMYDGF structural integrity was monitored and confirmed by NH-HSQC throughout the collection of NMR spectra.
Randomization	Not applicable, the study focuses on the structural characterization of one protein.
Blinding	Not applicable, the study focuses on the structural characterization of one protein.

Reporting for specific materials, systems and methods

We require information from authors about some types of materials, experimental systems and methods used in many studies. Here, indicate whether each material, system or method listed is relevant to your study. If you are not sure if a list item applies to your research, read the appropriate section before selecting a response.

Materials & experimental systems

n/a	Involved in the study	
☒	☐	Antibodies
☐	☒	Eukaryotic cell lines
☒	☐	Palaeontology
☒	☐	Animals and other organisms
☒	☐	Human research participants
☒	☐	Clinical data

Methods

n/a	Involved in the study	
☒	☐	ChIP-seq
☒	☐	Flow cytometry
☒	☐	MRI-based neuroimaging

Eukaryotic cell lines

Policy information about cell lines

Cell line source(s)	High Five cells (B85502, Thermo Fisher Scientific); Sf9 cells (11496015, Thermo Fisher Scientific)
Authentication	None of the cell lines used were authenticated.
Mycoplasma contamination	The cell lines were not tested for mycoplasma contamination.
Commonly misidentified lines (See ICLAC register)	*Name any commonly misidentified cell lines used in the study and provide a rationale for their use.*
